# The role of interleukin-1 beta in the pathophysiology of Schnitzler’s syndrome

**DOI:** 10.1186/s13075-015-0696-0

**Published:** 2015-07-22

**Authors:** Heleen D. de Koning, Joost Schalkwijk, Monique Stoffels, Johanna Jongekrijg, Joannes F. M. Jacobs, Eugène Verwiel, Hans J. P. M. Koenen, Frank Preijers, Dirk Holzinger, Irma Joosten, Jos W. M. van der Meer, Anna Simon

**Affiliations:** Departments of Dermatology, Radboud University Medical Center, PO Box 9101, 6500 HB Nijmegen, The Netherlands; Internal Medicine, Radboud University Medical Center, Nijmegen, The Netherlands; Laboratory Medicine – Laboratory for Medical Immunology, Radboud University Medical Center, Nijmegen, The Netherlands; Genetics, Radboud University Medical Center, Nijmegen, The Netherlands; Laboratory Medicine – Laboratory for Hematology, Radboud University Medical Center, Nijmegen, The Netherlands; Radboud Institute for Molecular Life Sciences, Nijmegen, The Netherlands; Nijmegen Center for Immunodeficiency and Autoinflammation, Nijmegen, The Netherlands; Department of Paediatric Rheumatology and Immunology, University Hospital Muenster, Muenster, Germany; Institute of Immunology, University Hospital Muenster, Muenster, Germany

## Abstract

**Introduction:**

Schnitzler’s syndrome (SchS) is a disabling autoinflammatory disorder, characterized by a chronic urticarial rash, an M-protein, arthralgia, and other signs of systemic inflammation. Anti-interleukin-1 (IL-1) beta antibodies are highly effective, but the pathophysiology is still largely unknown. Here we studied the effect of *in-vivo* IL-1 inhibition on serum markers of inflammation and cellular immune responses.

**Methods:**

Eight patients with SchS received monthly subcutaneous (s.c.) injections with 150 mg canakinumab for six months. Blood was drawn for measurement of serum markers of inflammation (12 times per patient) and for functional and phenotypic analysis of both freshly isolated and toll-like receptor (TLR)-ligand-stimulated peripheral blood mononuclear cells (PBMCs) (five times per patient). All data were compared to results of healthy controls.

**Results:**

IL-6 levels in serum and in lysates of freshly isolated PBMCs and serum myeloid-related protein (MRP8)/14 and S100A12 levels correlated with disease activity. *In vitro*, LPS stimulation resulted in higher IL-6 and IL-1 beta production in PBMCs from symptomatic SchS patients compared to healthy controls, whereas patient cells were relatively hyporesponsive to poly:IC and Pam3Cys. The mRNA microarray of PBMCs showed distinct transcriptomes for controls, symptomatic patients and anti-IL-1-treated patients. Numbers of T- and B-cell subsets as well as M-protein concentrations were not affected by IL-1 inhibition. Free light chain levels were elevated in 4 out of 8 patients.

**Conclusions:**

In conclusion, patient PBMCs are hyperresponsive to LPS, and clinical efficacy of IL-1 beta inhibition in patients with SchS is associated with *in-vivo* and *ex-vivo* suppression of inflammation. Interestingly, patient PBMCs showed divergent responses to TLR2/6, TLR3 and TLR4 ligands. Our data underscore that IL-1 beta plays a pivotal role in SchS.

**Electronic supplementary material:**

The online version of this article (doi:10.1186/s13075-015-0696-0) contains supplementary material, which is available to authorized users.

## Introduction

Schnitzler’s syndrome (SchS) is a chronic disabling autoinflammatory disorder, characterized by a chronic urticarial rash, a monoclonal component (M-protein), arthralgia and other signs and symptoms of systemic inflammation, with the long-term risk of development of a lymphoproliferative disorder [1–3]. The mean age of onset is 51 years, and a positive family history has never been reported. The etiology is unknown, but a pathophysiological clue has been provided by the efficacy of anti-interleukin-1 (IL-1) treatment [1, 4–11], and IL-1β inhibition in particular [12–14]. However, when IL-1 inhibition is discontinued, symptoms will rapidly return after stopping the IL-1 receptor antagonist (IL-1Ra) anakinra, or will gradually return after stopping canakinumab, a monoclonal anti-IL-1β antibody. This implies that the disease process continues upstream of IL-1β [13]. Also, whereas markers of systemic inflammation all normalize, M-protein concentrations remain unaffected during anakinra and canakinumab treatment [13].

Previous case reports showed that peripheral blood mononuclear cells (PBMCs) or monocytes from patients with symptomatic SchS produced more IL-1β and IL-6 upon lipopolysaccharide (LPS)-stimulation compared to control PBMCs [9, 15, 16]. Here, we studied the effect of several toll-like receptor (TLR) ligands on IL-1β, IL-6 and tumor necrosis factor alpha (TNFα) production by PBMCs of eight classical and variant SchS patients, including two variant patients with *NLRP3* mosaicism that were recently described [17]. Moreover, we performed these experiments, as well as serum cytokine measurements, leukocyte subset analyses and serum free light-chain analyses, on blood samples collected during a symptomatic episode, anakinra treatment, and at several time points during a trial with canakinumab [13] in order to investigate disease-specific characteristics and the effect of IL-1 on these markers.

We showed that the clinical efficacy of IL-1β inhibition in patients with SchS is associated with suppression of inflammation, and that TLR4 is involved in the enhanced IL-1β production. We also identified MRP8/14 and S100A12 as markers of disease activity in SchS.

## Methods

### Patients and patient samples

The study was approved by the local medical ethical committee of the Radboud university medical center, as the patients and controls were recruited there. Eight patients with SchS, either classical or variant type, and seventeen healthy controls that were age- and sex-matched as much as possible provided written informed consent. Patients stopped anakinra in order to enter the canakinumab trial and multiple blood samples were collected [13]. Polymorphonuclear cells (PMNs) and peripheral blood mononuclear cells (PBMCs) were isolated during anakinra treatment, during disease relapse after discontinuation of anakinra (symptomatic episode), 14 days and 6 months after the first monthly canakinumab injection, and upon disease relapse after discontinuation of canakinumab. At each time point, blood samples from a matched healthy donor control were collected too. B cells and T cells were isolated from blood samples collected during anakinra, canakinumab and during the symptomatic phase. Serum samples were also taken on those occasions, as well as at 3 and 7 days and then monthly after the first canakinumab injection.

### PBMC and polymorphonuclear cells (PMNs) processing

PBMCs were isolated from EDTA-blood using Ficoll-paque Plus (GE Healthcare, Eindhoven, The Netherlands) separation, and PMNs were isolated from the pellet by lysing erythrocytes with a hypotonic 155mM NH_4_Cl, 10 mM KHCO_3_ lysis buffer. For RNA isolation, 5 million cells of each sample were dissolved in 1 ml Trizol (Invitrogen, Bleiswijk, The Netherlands) and stored until further processing. For protein analysis, 6 million cells were lysed with a lysisbuffer (50 mM Tris (pH 7.4), 150 mM NaCl, 2 mM EDTA, 2 mM ethylene glycol tetraacetic acid (EGTA), 10 % glycerol, 1 % Triton X-100, 40 mM β-glycerophosphate, 50 mM sodium fluoride, and 200 mM sodium vanadate, supplemented with protease inhibitor cocktail (Roche, Mannheim, Germany)) and stored at −80 °C until measurement.

### PBMC culture

PBMCs isolated at the five indicated time points were also used for in vitro experiments. Cells from patients (N = 8) and controls (N = 17) were stimulated for 24 hours with LPS (TLR4 ligand, 0.1, 1, 10 ng/ml) (Sigma, St Louis, MO, USA; *Escherichia coli* serotype 055:B5, purified in our own laboratory), Pam3Cys (TLR2 ligand, 10 μg/ml) (EMC Microcollections, Tubingen, Germany), poly:IC (TLR3 ligand, 5 μg/ml) (InvivoGen, Toulouse, France), or no ligand. For IL-17 assays, cells were stimulated for 7 days with heat-killed (homemade) *Candida albicans* (10^6/ml). Supernatants were collected and stored at −80 °C. Cytokine concentrations in serum, supernatants and lysates were measured by means of enzyme-linked immunosorbent assay (ELISA): IL-1β (R&D, DY 201E, Minneapolis, MN, USA), IL-6 (Sanquin, M9316, Nijmegen, The Netherlands), TNF-α (R&D, DY210E), IL-17 (R&D, DY317E), MRP8/14 and S100A12 as previously described [18, 19].

### RNA extraction and quantitative real-time polymerase chain reaction (qPCR)

mRNA was extracted and first-strand cDNA was generated and amplified by means of qPCR as previously described [20]. Specific qPCR primers were designed with Primer Express 1.0 Software (Applied Biosystems) and purchased from Biolegio (Nijmegen, The Netherlands). By means of the comparative delta-DCt method, relative mRNA expression levels of all examined genes were calculated [21].

### Microarray

A microarray using the Illumina Direct Hybridization Assay (performed by ServiceXs, Leiden, The Netherlands) was performed on purified whole blood RNA samples from two patients with classical SchS and 1 IgG variant case with myeloid-restricted *NLRP3* mosaicism. Integrity of the RNA samples was confirmed by Eukaryote Total RNA Nano Bioanalyzer analysis. The microarray data were further analyzed by loading the log expression values into Partek Genomics Suite Software (Version 6.4; Partek, Inc., St Louis, MO, USA). Quantile normalization was performed including all arrays, to correct for large overall expression differences between the arrays. In addition, to adjust for the baseline expression level of each of the individual patients in the different subgroups, a correction for the factor ‘individual’ was performed, as one would do for batch correction, using the batch remove option from the software. On 1 August 2015, the data will be released online in the Gene Expression Omnibus [GSE70019].

### Flow cytometric analysis

#### *T-lymphocyte subsets*

To detect intracellular expression of the transcription factors Foxp3, RORγt and Tbet in CD3+CD4+ T cells, Ficoll-isolated PBMCs were first labeled with CD3(UCHT1)-ECD and CD4(SFCI12T4D11)-PC7 (Beckman Coulter), and subsequently fixed and permeabilized using Fix/Perm buffer (eBioscience), labeled with FoxP3(PCH101)-FITC (eBioscience), RORγt(AFKJS-9)-APC and T-bet(4B10)-PE (Santa Cruz Biotechnology, Santa Cruz, CA) and measured by five-color flow cytometry (FC500, Beckman Coulter). Data were analyzed using CXP software (Beckman Coulter). Isotype controls were used for gate settings.

#### *B-lymphocyte subsets*

Cells from heparinized blood were phenotypically analyzed in a 10-color MoAb conjugate combination using a Navios™ instrument with 10-color PMTs and three solid-state lasers (Beckman Coulter, Fullerton, FL). The list mode data files were further analyzed using Kaluza™ software (Beckman Coulter). In order to guarantee that the optics, laser, fluidics and fluorescence intensity were stable during all measurements calibration was performed using Flow Check Pro Fluorospheres (Beckman Coulter) and Cyto-Cal Multifluor + Violet Fluorescence Alignment Beads (Thermo Scientific, Fremont, CA, USA). After erythrocyte lysis (BD Pharm-Lyse, BectonDickinson) cells were washed with PBS with 1 % bovine serum albumin before being labeled with fluorochrome-conjugated mAbs. After incubation for 30 minutes at 4 °C in the dark, cells were washed twice to remove unbound antibodies and analyzed. For cell surface staining, the following mAbs were used: IgD-FITC, IgM-PE (both Dako, Denmark) and CD3-ECD, CD4-PECy5.5, CD27-PECy7, CD20-PacB, CD45-KromeOrange, CD56-APC, CD8-APC-Alexa Fluor700 and CD19-APC-Alexa Fluor750 (all Beckman Coulter, Marseille, France). Subsequently, the various lymphocyte subpopulations were analyzed on the flow cytometer using CD45/SSC to gate the lymphocyte population.

### M-protein and free light chain analysis

To detect and quantify the presence of an M-protein, agarose gel electrophoresis and immunofixation were performed on the Hydrasys (Sebia, Evry, France) according to the manufacturer’s protocol. Serum free light-chain analysis was performed on a BNII analyzer (Siemens, Marburg, Germany) using Freelite reagents (The Binding Site Ltd, Birmingham, UK) according to the manufacturer’s protocol.

### Statistical analysis

Repeated-measures analysis of variance (ANOVA) using SPSS v16.0 (SPSS Inc) was performed on the delta-cycle threshold (dCt) values of the qPCR data corrected for primer efficiency. dCt is the difference between the Ct of the target gene and the reference gene (*RPLP0*). The ELISA data were analyzed by one-way ANOVA with Bonferroni post hoc testing for the models. Fold changes and *p* values of the microarray data for the contrast, patients vs. controls, were calculated by conducting multifactorial ANOVA on the factor ‘treatment’.

## Results

### Proinflammatory cytokines and S100 proteins in serum, PBMCs and PMNs

**Fig. 1:**
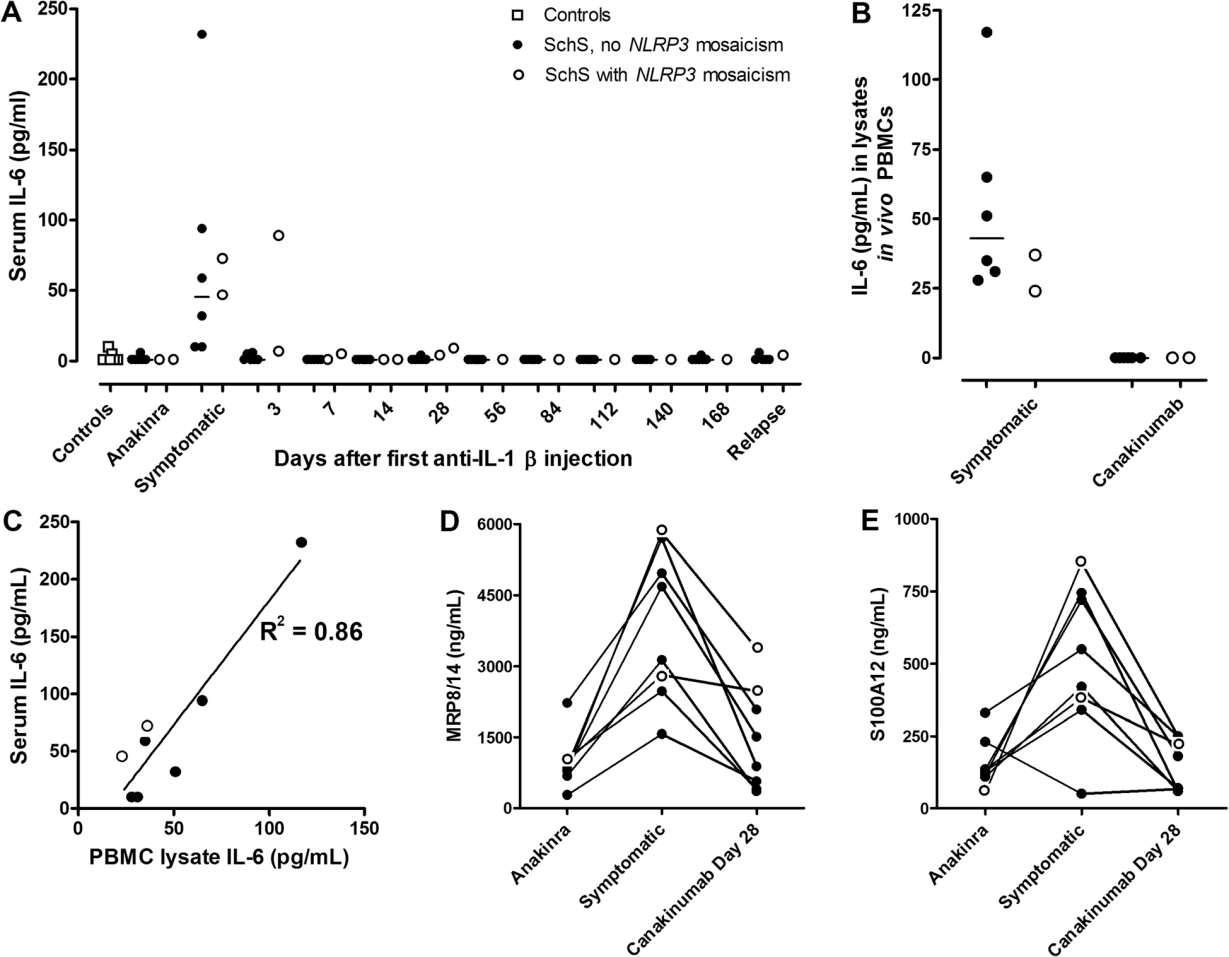
Serum concentrations of IL-6, myeloid-related protein (*MRP*)8/14 and S100A12, and peripheral blood mononuclear cell (*PBMC*)-associated IL-6 correlate with disease activity. **a** Serum IL-6 levels in controls, and in Schnitzler’s syndrome (*SchS*) patients with or without *NLRP3* mosaicism during anakinra (IL-1Ra) treatment, a symptomatic episode, at several time points during a canakinumab (anti-IL-1β antibody) trial, and during relapse after canakinumab withdrawal. **b** IL-6 concentration in lysates of freshly isolated PBMCs from SchS patients with or without *NLRP3* mosaicism. **c** Correlation between serum and cell-associated IL-6 concentrations in SchS patients. **d** Serum MRP8/14 and **e** S100A12 concentrations during anakinra treatment, a symptomatic episode, and 28 days after a single injection of canakinumab

Proinflammatory cytokine concentrations were measured in serum from patients under anakinra, during symptoms, and several time points upon initiating canakinumab treatment. Compared to controls, serum IL-6 was elevated in all patients during active disease, to become undetectable or very low during anakinra or canakinumab treatment (Fig. 1a). The presence or absence of *NLRP3* mosaicism did not make a difference. IL-17 was undetectable in all serum samples and TNF-α in most serum samples. Serum IL-1β was undetectable or very low in the samples taken during anakinra treatment and during active disease. Because binding to the administered antibodies probably interferes with detection, IL-1β could not be reliably measured during canakinumab treatment. Interestingly, clinical relapse was not associated with a rise in IL-6 concentrations. Cell-associated IL-6 concentrations (measured in lysates from PBMCs that were directly lysed after sampling) were also high during the symptomatic phase, and correlated with serum IL-6 concentrations (*R*^2^ = 0.86, Fig. 1b, c). We could not detect any IL-1β, IL-6 or IL-17 protein in neutrophil lysates.

Myeloid-related protein 8 (MRP8, or S100A8), MRP14 (S100A9) and S100A12 are known indicators of systemic inflammation [22–24]. Serum MRP8/14 and S100A12 concentrations corresponded with disease activity in all SchS patients as they were increased during symptomatic episodes and decreased during in vivo IL-1 inhibition (Fig. 1d, e). The clinical relapse in patient 8 and presence of minimal symptoms in patient 7 at day 28 after the first canakinumab dose were reflected by relatively high MRP8/14 and S100A12 concentrations, whereas serum IL-6 concentrations were only marginally increased (Figure a, d, e) [13]. MRP8/14 and S100A12 concentrations in active SchS (median 3,905 and 485 ng/ml, respectively) were higher than in reported healthy controls (median 340 ng/ml and 120 ng/ml, respectively) [24, 25], and in anakinra-treated patients (median 945 and 133 ng/ml, respectively) and canakinumab-treated patients (median 1,195 and 125 ng/ml, respectively). Interestingly, MRP8/14 serum levels during IL-1 inhibition were significantly higher than in the reported healthy controls. The patient data had considerable heterogeneity. The S100A12 data correlated with the MRP8/14 data (*R*^2^ = 0.92, 0.94 and 0.85, respectively).

As neutrophils were reported to possess IL-1β processing capacity [26], we measured cytokine mRNA expression levels of circulating PMN cells of controls and of patients with active disease or during IL-1 inhibition. During attacks and during treatment episodes we found significant expression of *IL1B* and *IL1RN* mRNA in neutrophils from two out of two SchS patients, and these levels were similar in two control samples (data not shown).

### Microarray of circulating PBMCs

As an unbiased approach, we performed a transcriptome analysis of RNA from PBMCs from three patients with SchS (one with the *NLRP3* mosaicism in myeloid cells) and three matched controls. To investigate the effect of IL-1 inhibition on gene expression, we analyzed patient samples drawn during symptomatic disease, and anakinra and canakinumab treatment. Assessment of unsupervised clustering of gene expression levels, normalized for the effect of the individual, revealed that controls, symptomatic patients, and treated patients were in separate clusters, with the largest difference between control and symptomatic patient samples (Additional file 1: Figure S1A).

Additional file 1: Figure S1B shows the clustering of the samples for the most significantly upregulated and downregulated genes in the symptomatic patients. Comparison of symptomatic patients to patients during IL-1 inhibition showed that *S100A12* and *IL-1B* are among the most differentially expressed genes during active disease. Both genes were significantly higher expressed in symptomatic patients than in anti-IL-1-treated patients and in controls, but mRNA levels during canakinumab or anakinra therapy did not differ significantly from those in controls. qPCR analysis confirmed the corresponding microarray data (Additional file 1: Figure S2).

### Differential production of proinflammatory cytokines by patient PBMCs stimulated with TLR ligands

**Fig. 2:**
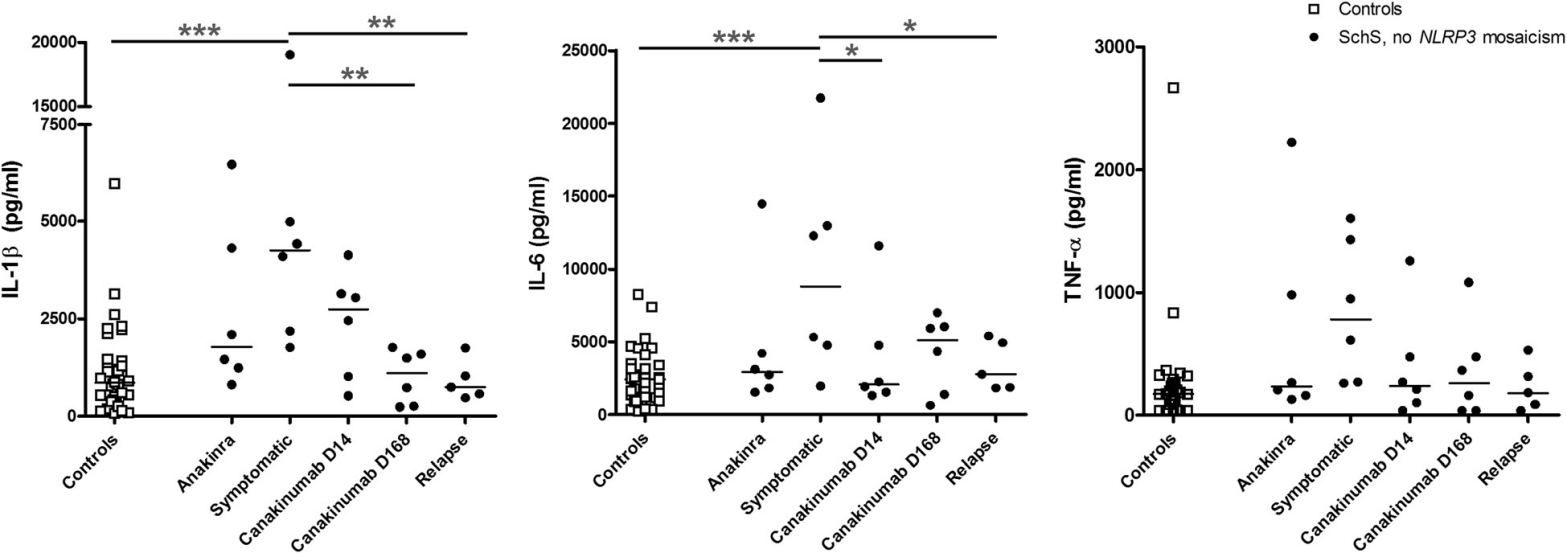
Lipolysaccharide (*LPS*)-induced production of IL-1β, IL-6, and TNFα in peripheral blood mononuclear cells (PBMCs) sampled during active disease and IL-1-blocking treatment. PBMCs of patients with Schnitzler’s syndrome (*SchS*) without *NLRP3* mosaicism were sampled during anakinra treatment, a symptomatic episode, at several time points during a canakinumab trial, and during relapse after canakinumab withdrawal. These PBMCs and those of healthy controls were stimulated with 1 ng/ml LPS for 24 hours, and supernatants were collected for ELISA of IL-1β, IL-6 and TNFα concentrations. Bars indicate median values. *p <0.05, **p <0.01, ***p <0.001

We assessed the proinflammatory response to LPS, Pam3Cys and poly:IC stimulation of control PBMCs and of patient PBMCs, sampled either in the symptomatic phase or during treatment with anakinra or canakinumab. As PBMCs of our two patients with myeloid mosaicism of *NLRP3* variants constitutively produced high levels of IL-1β and IL-6, whereas PBMCs of controls and patients without this mosaicism did not [17], we analyzed the results of the former separately (see below). In PBMCs of all patients sampled during a symptomatic episode, IL-1β, IL-6 and TNF-α production was higher than in healthy controls when the cells were exposed to 1 ng/ml LPS (Fig. 2, significance of the models, *p* = 0.002, 0.035, and 0.076, respectively). There was a clear dose response for cells exposed to 0.1 and 1.0 ng/ml LPS (data not shown). In controls and during treatment with canakinumab, the IL-1β production induced by LPS was lower (Fig. 2, *p* <0.001 and 0,003, respectively). LPS-induced IL-6 production was significantly elevated in PBMCs during the symptomatic phase compared to controls and during anti-IL-1β treatment (Fig. 2b, *p* = 0.04 and <0.001, respectively). Due to an outlier, this was significant for anakinra. The patients with the highest responses were all the classical IgM type and had a more severe phenotype than the others. No clear difference in proinflammatory cytokine production between patients and controls was found upon stimulation with LPS 10 ng/ml. In vitro addition of IL-1Ra only marginally inhibited IL-1β and IL-6 production (at the highest LPS concentration (10 ng/ml)) in PBMCs of all SchS patients (data not shown).

**Fig. 3:**
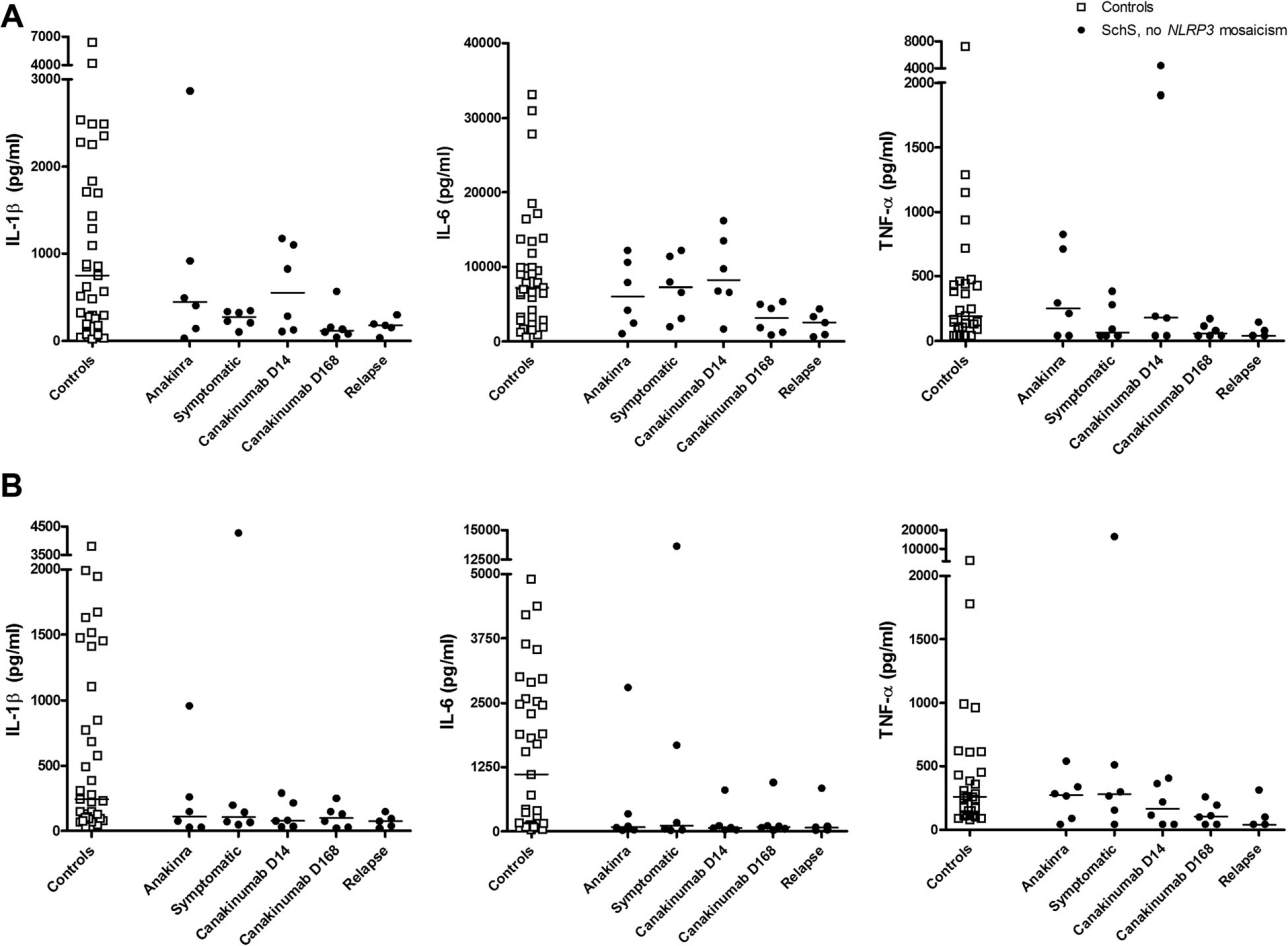
Pam3Cys- and poly:IC-induced production of IL-1β, IL-6, TNFα in peripheral blood mononuclear cells (PBMCs) sampled during active disease and IL-1-blocking treatment. PBMCs of patients with Schnitzler’s syndrome (*SchS*) without *NLRP3* mosaicism were sampled during anakinra treatment, a symptomatic episode, at several time points during a canakinumab trial, and during relapse after canakinumab withdrawal. These PBMCs and those of healthy controls were stimulated with Pam3Cys 10 μg/ml (**a**), or poly:IC 5 μg/ml (**b**) for 24 hours, and supernatants were collected for ELISAs of IL-1β, IL-6 and TNFα concentrations. Bars indicate median values

When cells of SchS patients and controls were exposed to Pam3Cys, production of IL-1β and IL-6 was significantly lower in the patients than in controls. On exposure to poly:IC this was not significant, but there was a clear trend (Pam3Cys IL-1β pooled patient data versus controls *p* = 0.006; Pam3Cys IL-6 pooled patient data versus controls *p* = 0.037; poly:IC IL-1β pooled patient data versus controls *p* = 0.058; poly:IC IL-6 pooled patient data versus controls *p* = 0.115). Interestingly, the treatment condition did not affect this production (Fig. 3).

In PBMCs of the two patients with myeloid *NLRP3* mosaicism (patients 7 and 8), high baseline (hence unstimulated) IL-1β and IL-6 production was seen, as previously reported. Addition of IL-1Ra in vitro inhibited this production, pointing to autocrine or paracrine cytokine production mediated by IL-1 [17]. In one of these patients (patient 7), the unstimulated production of IL-6 and IL-1β was lower during IL-1 blocking treatment than during the symptomatic phase. LPS, Pam3Cys and poly:IC induced production of both cytokines during the symptomatic episode in this patient. This was also reduced during both anakinra and canakinumab treatment. The data for patient 8 were inconsistent (Additional file 1: Figure S3). However, in both patients, the production of IL-1β and IL-6 was dose-dependent over a range of 0.1 to 10.0 ng/ml LPS (data not shown). After 7 days stimulation with heat-killed *Candida albicans*, IL-17 production by PBMCs from patients and controls was similar, irrespective of the disease status of the patients (data not shown).

### T and B lymphocyte analyses

Circulating T and B lymphocyte subsets were analyzed by means of flow cytometry during active disease and under anakinra and canakinumab treatment. Numbers of regulatory T cells and expression levels of Foxp3 were similar in patients during different treatment settings and in controls, and there was no difference in RORyT+ cells (Additional file 1: Figure S4). Regulatory T cell suppressor function as studied in a co-culture suppression assay appeared unaffected - we found no difference between patient and control cells (n = 1; data not shown). There were also no B-lymphocyte subset changes during the different treatment settings (Additional file 1: Figure S5).

### M-protein and serum free light-chain concentrations

In our patients, the serum concentration of the M-protein, the diagnostic hallmark of SchS, was not related to disease activity. IL-1 inhibition did not affect paraprotein concentrations in our patients either during several years of anakinra treatment or during canakinumab treatment for 6 months. Moreover, in our two patients with the most severe phenotype and in which *NLRP3* somatic mosaicism was found in myeloid cells, only unquantifiable IgG kappa was found (Table 1).

**Table 1:** M-protein and serum free light-chain levels during IL-1 inhibition

Patient	Sex	Treatment	M-protein		Serum free light-chains		
number			subtype	g/L	kappa (mg/l)	lambda (mg/l)	Ratio κ / λ
1	M	Anakinra	IgMκ & IgMλ	10.1	14.4	**46.8**	0.31
		None (Day 4 after anakinra withdrawal)		7.7	14.7	**53.9**	0.27
		Canakinumab day 28		10.6	18.6	**51.0**	0.36
		Relapse (post-canakinumab)		9.3			
2	F	None (pre-anakinra)	IgGκ	6.8	**88.3**	12.4	**7.12**
		Anakinra		5	**59.9**	9.4	**6.35**
		None (day 5 after anakinra withdrawal)		4.1	**69**	13.5	**5.11**
		Canakinumab Day 28		4.3	**59.1**	11.6	**5.09**
		Relapse (post-canakinumab)		3.7	**78.4**	12.2	**6.43**
3	M	None (day 5 after anakinra withdrawal)	IgMκ	2.7			
		Canakinumab day 28		3.5	19.1	19.8	0.96
4	M	Anakinra	IgGκ	2.9			
		None (day 5 after anakinra withdrawal)		2.8			
		Canakinumab day 28		3.2	14	10.3	1.36
		Relapse (post-canakinumab)		2.5			
5	M	Anakinra	IgMκ	4.4			
		None (day 6 after anakinra withdrawal)		4.5	19.4	12.4	1.56
		Canakinumab day 28		5			
		Relapse (post-canakinumab)					
6	M	Anakinra	IgMκ	6.8	**25.5**	20.6	1.24
		None (day 4 after anakinra withdrawal)		6.1	**29.2**	15.8	1.85
		Canakinumab day 28		5.6	**29.3**	17.9	1.64
		Relapse (post-canakinumab)		6.3			
7	F	Anakinra	IgGκ	n.d.			
		None (day 3 after anakinra withdrawal)		n.d.	15.3	15.1	1.01
		Canakinumab day 28		n.d.			
		Relapse (post-canakinumab)		n.d.			
8	M	Anakinra	IgGκ	n.d.	17	13.5	1.26
		None (day 5 after anakinra withdrawal)		n.d.	**26.9**	18.7	1.44
		Canakinumab day 14		p.n.q.	18.4	18.1	1.02
		Canakinumab day 28 (relapse)		p.n.q.	**23.9**	23.2	1.03

Free light-chains in the serum were measured with the Freelight assay, reference values: serum free kappa chains (3.3−19.4 mg/l); serum free kappa chains (5.7−26.3 mg/l); ratio kappa/labda light-chains (0.26−1.65). Abnormal values are indicated in bold. *n.d.* not detectable; *p.n.q.* present but not quantifiable

The ratio of serum kappa and lambda free light-chain levels was reported to be a prognostic factor for disease progression in multiple myeloma and other monoclonal gammopathies [27–29]. Hence, we measured this ratio in eight patients, in two of whom it was abnormal, especially in a female patient with strongly elevated kappa light-chain levels. In these and two other patients who had increased free light-chain levels, we tested free light-chains during symptoms and during treatment with IL-1 inhibition. We observed no significant changes in serum free light-cchain concentrations in symptomatic SchS patients versus patients in remission due to IL-1 inhibition (Table 1).

## Discussion

Here, we described the inflammatory response during the symptomatic phase of SchS, during treatment with either anakinra or canakinumab, and during relapse after canakinumab withdrawal. During the symptomatic phase, the circulating concentrations of IL-6 were elevated, as were the protein concentrations of IL-6 and mRNA levels of IL-1β in circulating PBMCs. In this phase there was also augmented LPS-induced production of IL-1β and IL-6. Finally, the MRP8/14 and S100A12 concentrations in serum and S100A12 mRNA levels in PBMCs were elevated. All of these were normalized during treatment with either IL-1Ra or anti-IL-1β antibodies, and both therapies led to a shift of the PBMC transcriptome towards the mRNA signature of healthy controls. Clinical relapse several months following canakinumab withdrawal was not associated with a rise in IL-6 serum concentrations, nor with increased ex vivo cytokine production by PBMCs. Interestingly, irrespective of the treatment condition, PBMCs from SchS patients produced less IL-1β and IL-6 when exposed to Pam3Cys or poly:IC when compared to controls.

Taken together, our data point to an IL-1β-driven disorder, which is in line with the clinical efficacy of IL-1β inhibition and with the few reports on in vitro findings. Recently, we reported two variant SchS patients with *NLRP3* mosaicism in the myeloid lineage, in whom PBMCs produced high constitutive levels of IL-1β and IL-6, which was abolished by in vitro addition of IL-1Ra [17]. Previously, a few SchS cases were described in which PBMCs or monocytes from symptomatic patients produced more IL-1β and IL-6 upon LPS stimulation compared to control PBMCs [9, 15, 16], and that this could be reversed by in vivo anakinra treatment [15]. Spontaneous IL-1β production by PBMCs was present in one more patient [16], but absent in others [15].

In our patients with *NLRP3* mosaicism, the hyperproduction of IL-1β is probably due to an overactive NLRP3 inflammasome. In those without this genetic defect the trigger of the enhanced IL-1β production is still unclear. The increased IL-1β concentrations in turn lead to production of IL-6 and an autocrine or paracrine production of more IL-1β. The latter became clear from the striking reduction in spontaneous IL-1β and IL-6 production by PBMCs from the two *NLRP3* mosaic patients when IL-1Ra was added in vitro [17], the reduced IL1B mRNA levels in circulating PBMCs during in vivo IL-1 inhibition, and the lower LPS-induced IL-1β and IL-6 production in PBMCs sampled during treatment.

The increased downstream production of cytokines leads to an enhanced acute phase response with elevation of C-reactive protein (CRP). The amount of IL-6, which is readily measurable in the circulation, is probably responsible for the fever and other signs of systemic inflammation.

How should we envisage the lack of a rise in serum IL-6 and the still downregulated cytokine production when the patients relapsed several months after canakinumab withdrawal? Probably the best explanation is that the relapse is compartmentalized in its early phase, possibly at the level of the skin, which is continuously exposed to pathogen-associated molecular patterns as well as endogenous ligands of pattern recognition receptors (PRRs). Indeed, PRRs were implicated in the pathophysiology of other inflammatory skin diseases, such as AIM2 (absent in melanoma 2) and dectin-1 in psoriasis [30–32]. The IL-1β positive mast cells we recently identified in SchS skin might not only be involved in the chronic urticaria (de Koning et al., submitted), but also in the induction of systemic inflammation.

As the triggers of the IL-1β production are currently unclear, it is of great interest that we find that TLR4 plays a clear role - and not TLR2, TLR3 and TLR6. It implies that either exogenous TLR4 ligands (such as LPS) or putative endogenous TLR4 ligands (like heat-shock proteins, minimally modified LDL, HMGB1, SAA3, MRP8/14, and S100A12 [33, 34]) function as triggers for the attacks. Especially, MRP8/14 and S100A12 are interesting in this regard, as serum levels are associated with disease activity in SchS. Several in vitro studies and mouse models of other inflammatory skin diseases have demonstrated a role for TLR4, e.g., nickel-induced allergic contact dermatitis and graft versus host disease [32]. Intriguingly, expression of both TLR4 and NLRP3 mRNA is extremely low in healthy epidermis, which one might consider a protective strategy preventing continuous stimulation by constituents of the microbiome, for example [31]. We detected a relatively decreased responsiveness to Pam3Cys (TLR2/6 ligand) and poly:I:C (TLR3 ligand) of the SchS patient PBMCs (sampled both during symptoms and anti-IL-1 treatment) compared to control PBMCs. To our knowledge, such divergent responses to TLR2, TLR3, TLR6 and TLR4 ligands have not been reported in inflammatory diseases before. We speculate that the relative hyporesponsiveness to TLR2 and TLR3 ligands might be a protective mechanism in response to the enhanced proinflammatory response to TLR4 ligands.

Our findings of elevated spontaneous cytokine production are reminiscent of findings in patients with the cryopyrin-associated periodic syndrome CAPS in which systemic inflammation is caused by activating *NLRP3* mutations. CAPS patient PBMCs constitutively produce IL-1β, and treatment with IL-1Ra results in both a dramatic clinical improvement and substantive downregulation of LPS-induced IL-1β secretion by the patients’ cells in vitro [35]. Typically, the enhanced proinflammatory response to lower LPS concentrations that we observed in SchS is also seen in other autoinflammatory diseases, whereas at the relatively high concentration of 10 ng/ml, the difference is much smaller or absent [36].

Hence, current and previous findings suggest that an inflammasome is primed in PBMCs in SchS as in CAPS, which explains a substantial IL-1β release in the absence of the second hit that is usually required. Also, the relative hyporesponsiveness to TLR2/6 and TLR3 agonists (in this study) and ATP [16], and low IL18 mRNA levels in monocytes despite high IL-18 serum levels [37] suggest the presence of several negative feedback mechanisms.

We previously reported that in the two patients with *NLRP3* mosaicism in the myeloid cell lineage, high constitutive IL-1β and IL-6 production by PBMCs was blocked by in vitro addition of IL-1Ra [17]. Here we show that this high baseline production was not impaired or was only partially impaired while patients were treated with IL-1 inhibitors. This implies ongoing activation of IL-1β, and may explain why these two patients had the most severe phenotypes and their disease quickly relapsed upon cessation of anti-IL-1 treatment [13]. TLR2/6 and TLR4 ligands induced the production of IL-1β and IL-6 in these two patients, but a TLR3 ligand did not.

In this study we additionally monitored S100 proteins, B cell and T cell subsets, M-proteins and serum free light-chains. S100A8/A9 (MRP8/14) and S100A12 are secreted after activation of phagocytes via a so-called alternative secretory pathway and cause strong proinflammatory effects on phagocytes and endothelial cells in vitro [24]. S100A8 and S100A9 and S100A12 have been shown to act as endogenous TLR-4 ligands and binding of S100 proteins to TLR-4 consequently induces NFκB expression via the MyD88-dependent pathway [33, 34]. Besides their pathophysiological role as DAMPs S100 proteins are regarded as markers of systemic inflammation [22–24] and especially in CAPS, both MRP8/14 and S100A12 levels mirrored disease activity, and were suggested as a sensitive marker even for subclinical disease [23, 38]. In CAPS patients responding to canakinumab treatment S100A8/A9 serum levels already dropped to the range of normal controls within 8 days [25].

In our study, S100A12 mRNA levels were significantly higher in circulating PBMCs from symptomatic patients than in PBMCs from treated patients and controls, and serum protein levels of both MRP8/14 and S100A12 correlated with disease activity. In a previous report, serum S100A12 protein levels did not correlate with disease activity in SchS, which might be related to the higher levels found in that study [5]. In most patients, MRP8/14 levels were higher than in healthy controls, even when they were asymptomatic under anakinra or canakinumab treatment [24]. Thus, MRP8/14 and S10012 levels are markers of disease activity in SchS. The patient data showed considerable heterogeneity, and in several patients persistent elevated MRP8/14 levels under treatment were found compared to healthy controls, as was previously reported in CAPS patients [38]. This may indicate subclinical disease activity that is not detected by CRP or IL-6 measurements.

Previously, an increase in transitional B cells, decrease in switched-memory B cells and low levels of peripheral blood plasma cells were reported in a patient with SchS when compared to healthy controls. IL-1Ra treatment had no effect on the patient’s B lymphocytes or the IgM M-protein [39]. Our analyses on T cell and B cell subsets showed no differences between active disease and anti-IL-1 treatment, neither were there any differences in T cell subsets between patients and controls. We do not know if long-term IL-1 inhibition would affect the T and B cell compartments.

An M-protein is one of the diagnostic hallmarks of Schs. We demonstrated that it is not a marker for disease activity as the serum concentration of the M-protein was not affected by IL-1 inhibition. Moreover, in our two most severely affected patients, only unquantifiable IgG kappa was found. Still, it cannot be excluded that long-term anti-IL-1 treatment could halt a progressive increase in M-protein concentration in view of the B cell activating property of IL-1β (the longest treatment duration of SchS patients on continuous anti-IL-1 treatment is currently 8 years). Moreover, long-term follow up of many SchS patients is needed to determine if IL-1 inhibition can prevent progression to a lymphoproliferative disorder. Indeed, in some patients with smoldering or indolent multiple myeloma, who were at risk of progression to active myeloma, concomitant treatment with IL-1Ra and dexamethasone was found to decrease the myeloma proliferative rate [40].

The ratio of serum immunoglobulin kappa and lambda light-chain levels is a prognostic factor for multiple myeloma disease progression [27, 28]. No such correlation was seen in our SchS patient cohort, but in one case, the highest free light-chain level was found prior to starting treatment with IL-1 inhibition. More measurements comparing pre- and post-IL-1 inhibition serum light-chain levels are needed to examine a possible association. We conclude that both the intact M-protein and the free light-chain concentrations are stable biomarkers in SchS patients, and are not affected by disease status or therapeutic intervention.

## Conclusions

Clinical efficacy of IL-1β inhibition in patients with SchS is associated with suppression of inflammation. We identified MRP8/14 and S100A12 as markers of disease activity in SchS. Our collective data underscore that IL-1β plays a pivotal role in SchS, and that TLR4 is involved in the enhanced IL-1β production. Future studies should be directed at the mechanism behind the differential responses to different TLR ligands and what drives the pivotal TLR4 response in the disease process.

## Additional file

Additional file 1: Figure S1. Microarray data. A Unsupervised clustering of microarray data of peripheral blood mononuclear cells (PBMCs) from healthy controls (*ctr*), Schnitzler’s syndrome patients (*pat*) with active disease, and Schnitzler’s syndrome patients treated with anakinra (*ana*) or canakinumab (*can*). B Clustering of the samples for the most significantly upregulated and downregulated genes in the symptomatic patients. Figure S2 Quantitative polymerase chain reaction (qPCR) validation of IL1B and S100A12 mRNA expression. IL1B and S100A12 mRNA expression in PBMC from controls (N = 18) and patients (N = 8) during anakinra treatment, canakinumab treatment, during symptoms, or during relapse after canakinumab withdrawal were evaluated by means of qPCR assays. Figure S3 Spontaneous and TLR2/6-/3-/4-stimulated production of IL-1β and IL-6 in PBMCs of NLRP3 mosaic patients. BMCs of patients with Schnitzler’s syndrome (SchS) with NLRP3 mosaicism that were sampled during a symptomatic episode, anakinra treatment, and canakinumab treatment were exposed to lipolysaccharide (LPS) 1 ng/ml, Pam3Cys 10 μg/mL poly:IC 5 μg/ml, or no stimulus for 24 hours, and supernatants were collected for ELISA of IL-1β (A), and IL-6 concentrations (B). Figure S4 No correlation treatment status with absolute numbers in several T-cell subsets. Several T-cell subsets were assessed by means of fluorescence-assisted cell-sorting during a symptomatic episode, during anakinra or canakinumab treatment, and at the time of relapse after canakinumab withdrawal. FOXP3+ cells, RORγt+ cells and CD25+CD127- (T regulatory) cells were measured in healthy controls and in patients with SchS during canakinumab treatment (N = 8) or relapse (N = 4). Figure S5 No correlation treatment status with absolute numbers in several B-cell subsets. Several B-cell subsets were assessed by means of fluorescence-assisted cell-sorting during a symptomatic episode, during anakinra or canakinumab treatment, and at the time of relapse after canakinumab withdrawal.

## Abbreviations

ANOVA: analysis of variance
CRP: C-reactive protein
dCt: delta-cycle threshold
ELISA: enzyme-linked immunosorbent assay
IL-1: interleukin-1
IL-1Ra: interleukin-1 receptor antagonist
LPS: lipopolysaccharide
mAB: monoclonal antibody
MRP: myeloid-related protein
PBMC: peripheral blood mononuclear cell
PBS: phosphate-buffered saline
PRR: pattern recognition receptor
PMN: polymorphonuclear cell
s.c.: subcutaneous
SchS: Schnitzler’s syndrome
TLR: toll-like receptor
TNFα: tumor necrosis factor alpha
