# Supplementary material for: The role of interleukin-1 beta in the pathophysiology of Schnitzler’s syndrome
Source: Arthritis Res Ther. 2015 Jul 22;17(1):187. doi: 10.1186/s13075-015-0696-0 (PMC4511239; doi:10.1186/s13075-015-0696-0)

**Additional file 1**

**The role of interleukin-1 beta in the pathophysiology of Schnitzler’s syndrome**

Heleen D. de Koning, et al.

**Figure S1A.**

Unsupervised clustering of microarray data of peripheral blood mononuclear cells from healthy controls (ctr), Schnitzler’s syndrome patients with active disease (nil = no treatment), and Schnitzler’s syndrome patients treated with anakinra (IL-1 receptor antagonist, ana) or canakinumab (anti-IL-1β antibody, can). Controls and patients; and symptomatic patients and treated patients cluster separately.


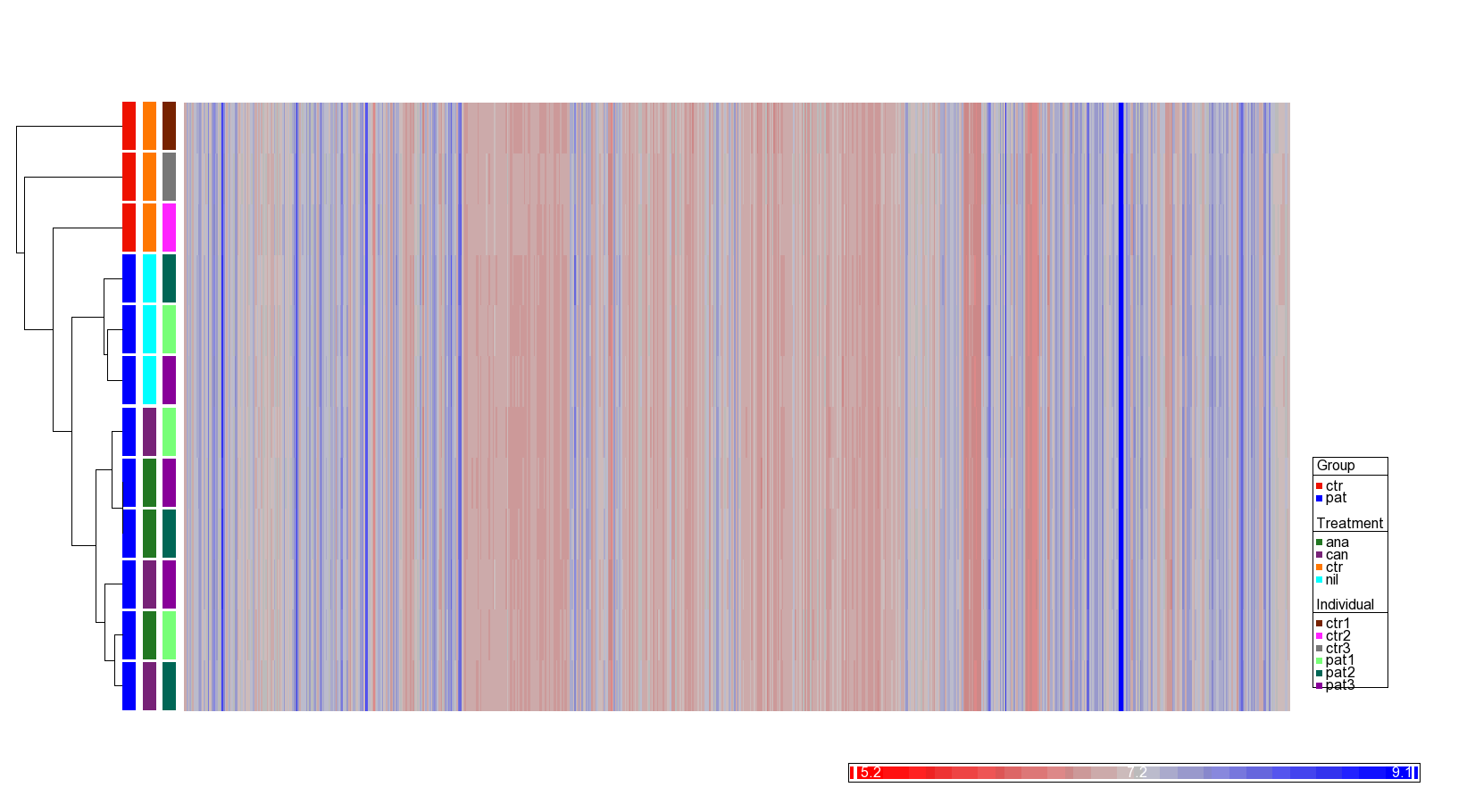

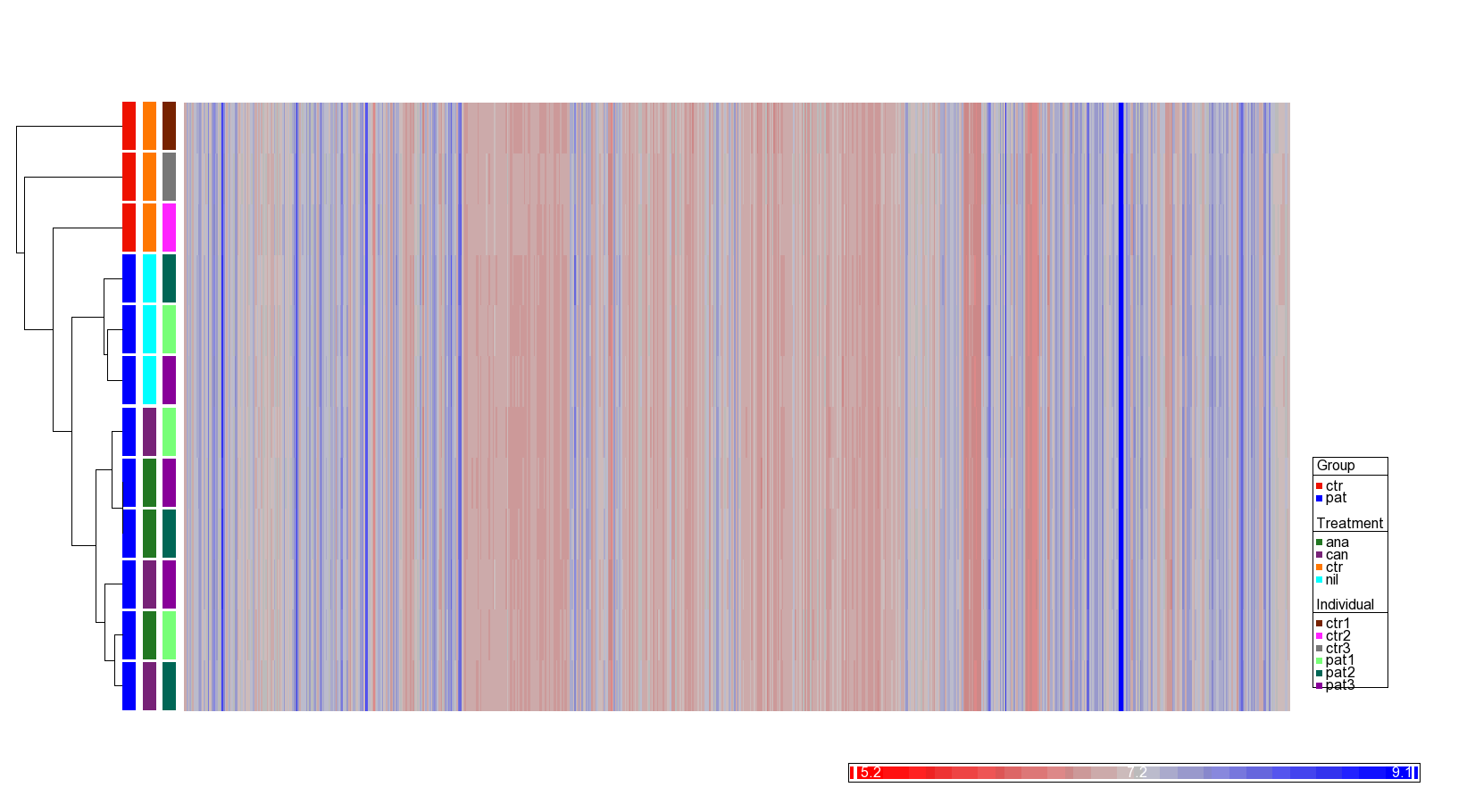


**Figure S1B.**

Clustering of the samples for the most significantly upregulated and downregulated genes in the symptomatic patients.


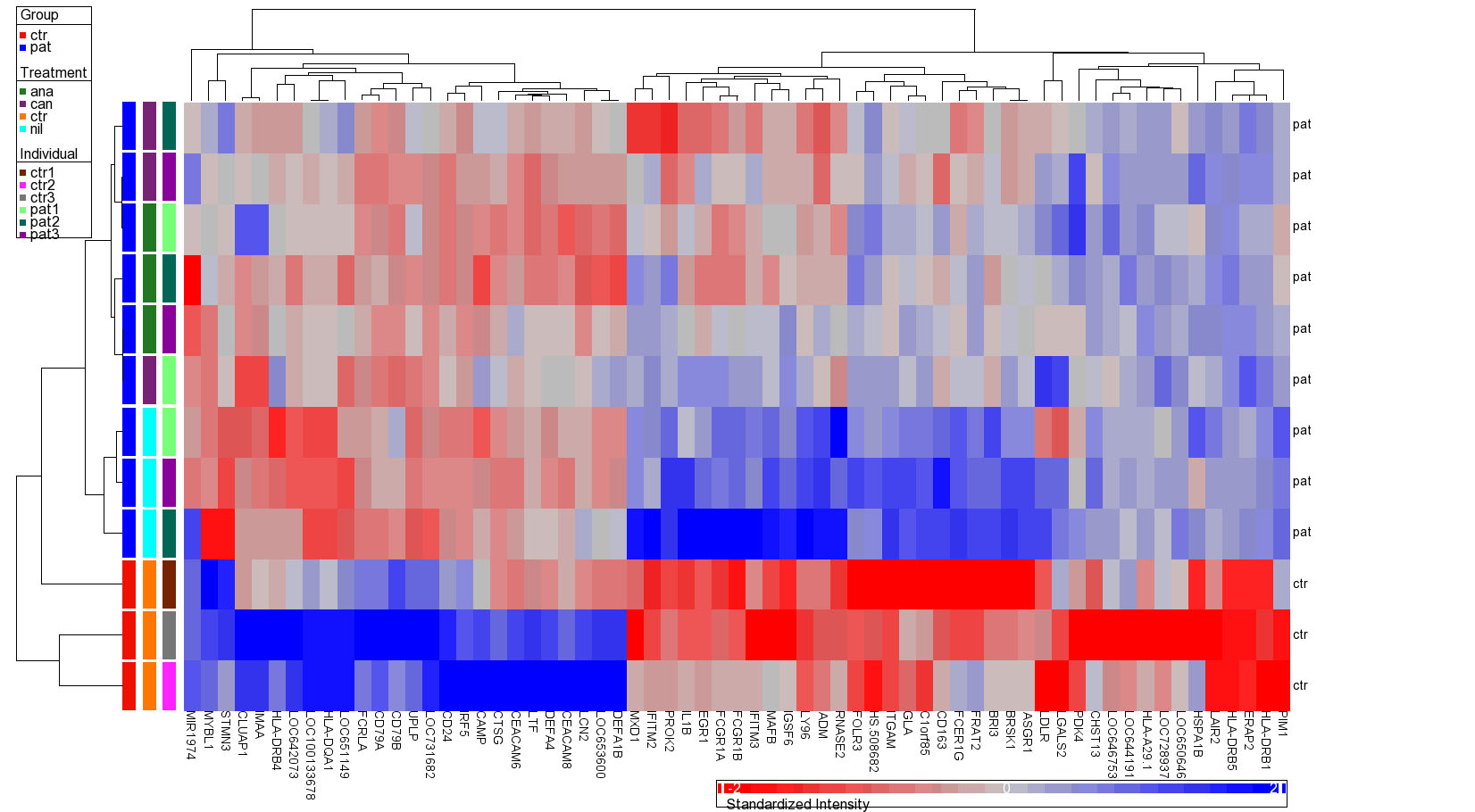


**Figure S2. qPCR validation of IL1B and S100A12 mRNA expression**

IL1B and S100A12 mRNA expression in PBMCs from controls (N=18) and patients (N=8) during anakinra treatment, canakinumab treatment, during symptoms, or during relapse after canakinumab withdrawal were evaluated by means of quantitative polymerase chain reaction assays. Quantities are depicted relative to the mean of controls. * P <0,05, ** P<0,01. Bars indicate mean +/- SEM.


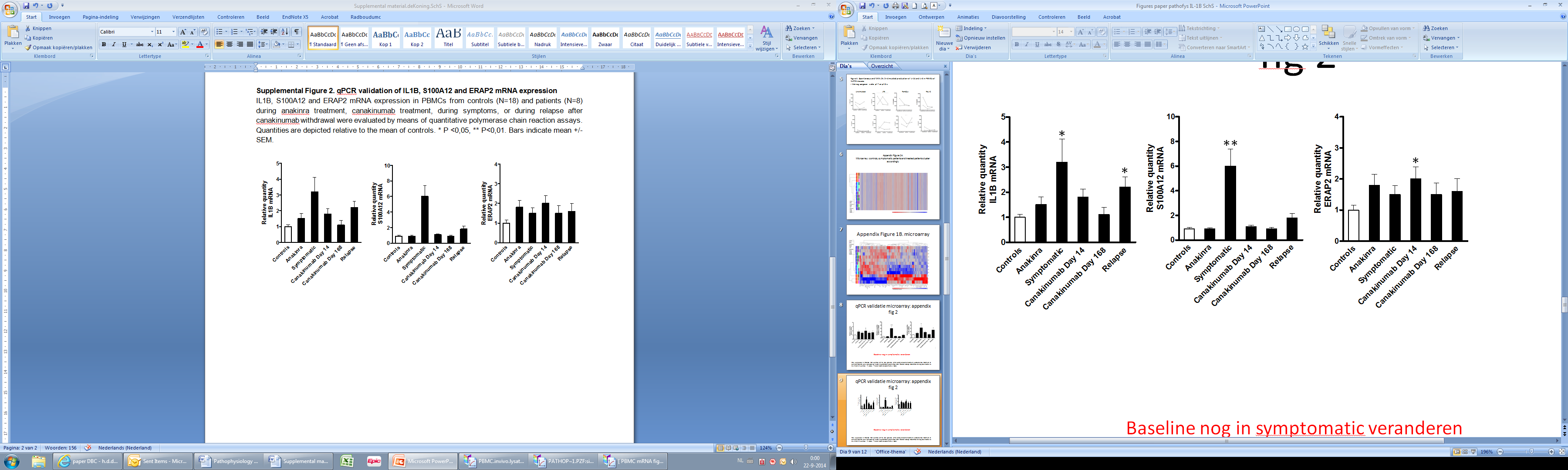


**Figure S3. Spontaneous and TLR2/6-/3-/4-stimulated production of IL-1β and IL-6 in PBMCs of NLRP3 mosaic patients**

PBMCs of patients with Schnitzler’s syndrome with NLRP3 mosaicism that were sampled during a symptomatic episode, anakinra treatment, and canakinumab treatment were exposed to LPS 1 ng/mL, Pam3Cys 10 µg/mL poly:IC 5 µg/mL, or no stimulus for 24 hours, and supernatants were collected for ELISAs of **A.** IL-1β, and **B.** IL-6 concentrations.

**
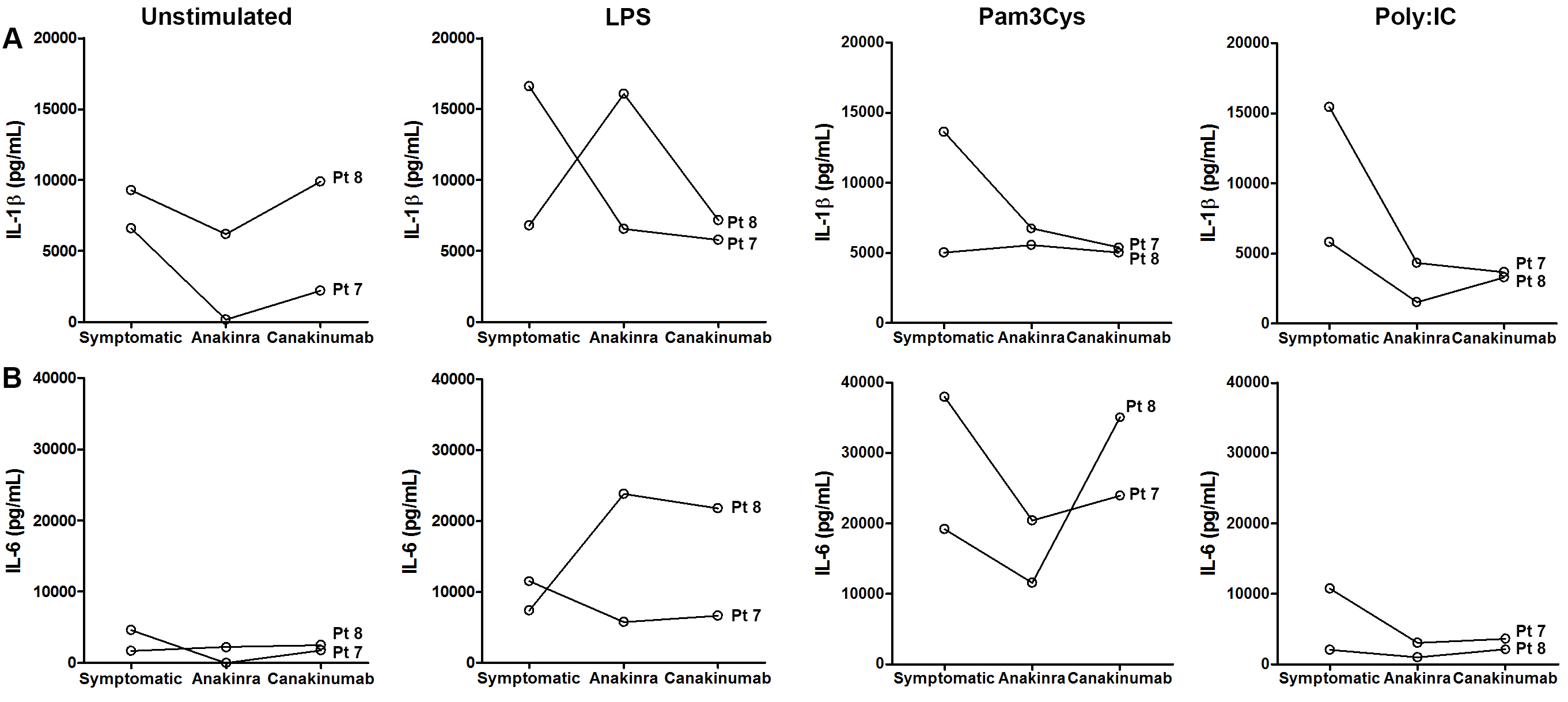
**

**Figure S4. No correlation treatment status with absolute numbers in several T-cell subsets**

Several T-cell subsets were assessed by means of fluorescence-assisted cell-sorting during a symptomatic episode, during anakinra or canakinumab treatment, and at the time of relapse after canakinumab withdrawal. FOXP3+ cells, RORγt+ cells and CD25+CD127- (T regulatory) cells were measured in healthy controls and in Schnitzler’s syndrome patients (SchS) during canakinumab treatment (N=8) or relapse (N=4).


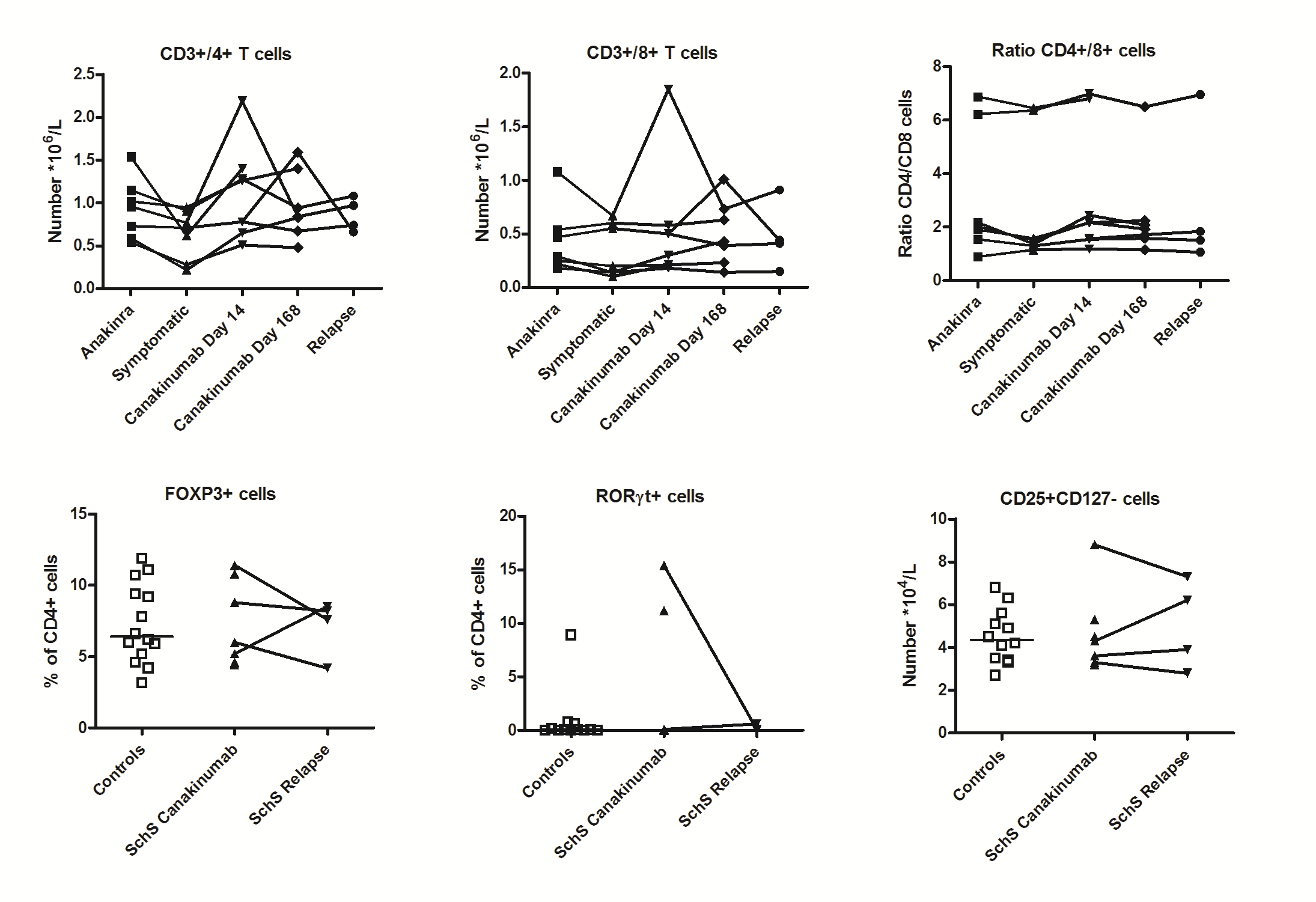


**Figure S5. No correlation treatment status with absolute numbers in several B-cell subsets**

Several B-cell subsets were assessed by means of fluorescence-assisted cell-sorting during a symptomatic episode, during anakinra or canakinumab treatment, and at the time of relapse after canakinumab withdrawal. In patient 3 (IgMk stable 3.4mg/L, died in accident before relapse occurred) higher IgM+ cells were present, especially on Day 168; not in others even though some had higher IgM M-component concentrations.


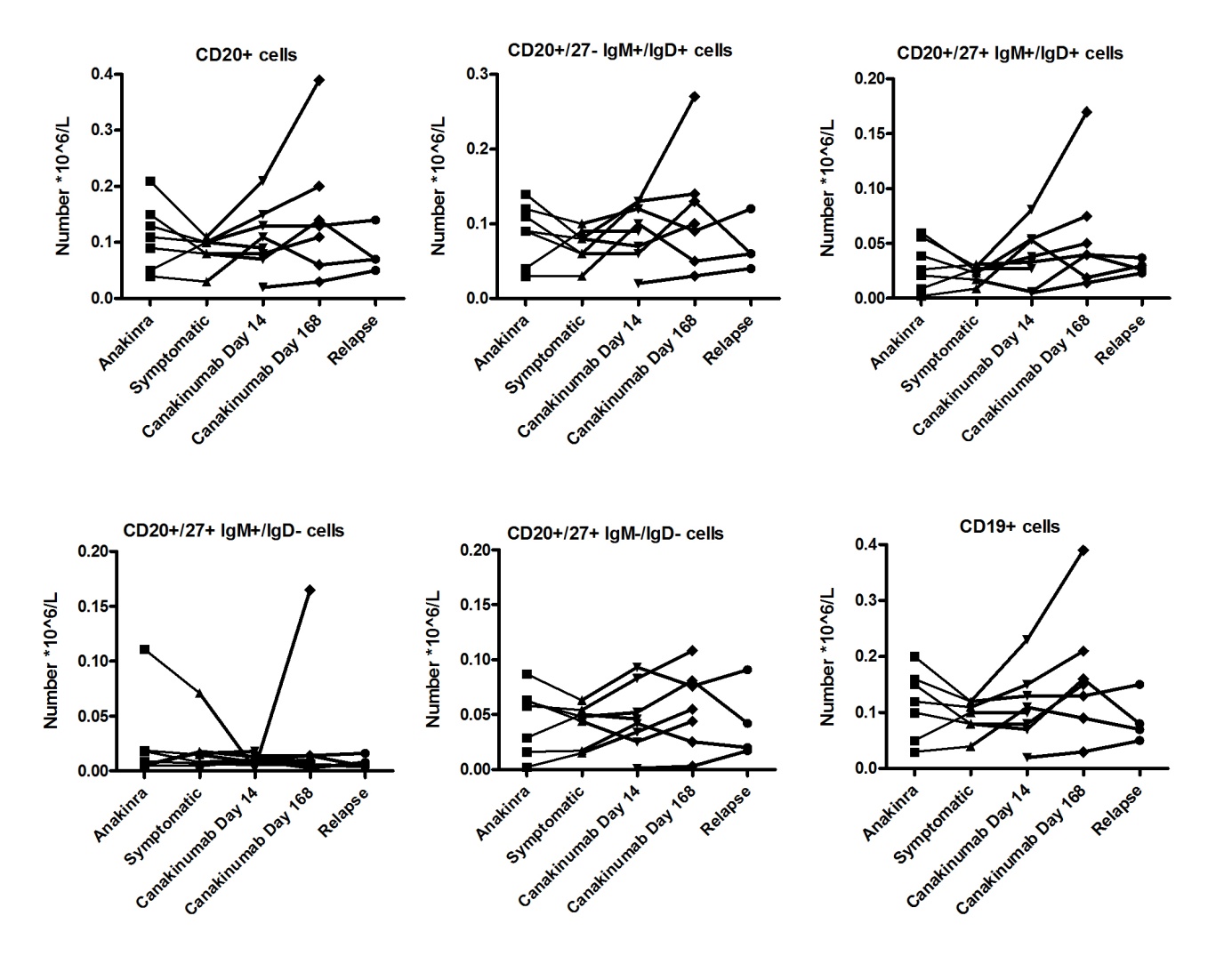

Supplement: Additional file 1: Figure S1. — Microarray data. A Unsupervised clustering of microarray data of peripheral blood mononuclear cells (PBMCs) from healthy controls (ctr), Schnitzler’s syndrome patients (pat) with active disease, and Schnitzler’s syndrome patients treated with anakinra (ana) or canakinumab (can). B Clustering of the samples for the most significantly upregulated and downregulated genes in the symptomatic patients. Figure S2 Quantitative polymerase chain reaction (qPCR) validation of IL1B and S100A12 mRNA expression. IL1B and S100A12 mRNA expression in PBMC from controls (N = 18) and patients (N = 8) during anakinra treatment, canakinumab treatment, during symptoms, or during relapse after canakinumab withdrawal were evaluated by means of qPCR assays. Figure S3 Spontaneous and TLR2/6-/3-/4-stimulated production of IL-1β and IL-6 in PBMCs of NLRP3 mosaic patients. BMCs of patients with Schnitzler’s syndrome (SchS) with NLRP3 mosaicism that were sampled during a symptomatic episode, anakinra treatment, and canakinumab treatment were exposed to lipolysaccharide (LPS) 1 ng/ml, Pam3Cys 10 μg/mL poly:IC 5 μg/ml, or no stimulus for 24 hours, and supernatants were collected for ELISA of IL-1β (A), and IL-6 concentrations (B). Figure S4 No correlation treatment status with absolute numbers in several T-cell subsets. Several T-cell subsets were assessed by means of fluorescence-assisted cell-sorting during a symptomatic episode, during anakinra or canakinumab treatment, and at the time of relapse after canakinumab withdrawal. FOXP3+ cells, RORγt+ cells and CD25+CD127- (T regulatory) cells were measured in healthy controls and in patients with SchS during canakinumab treatment (N = 8) or relapse (N = 4). Figure S5 No correlation treatment status with absolute numbers in several B-cell subsets. Several B-cell subsets were assessed by means of fluorescence-assisted cell-sorting during a symptomatic episode, during anakinra or canakinumab treatment, and at the time of relapse after canakinumab with [file 13075_2015_696_MOESM1_ESM.doc]
